# Supplementary material for: Rapid chromosome evolution and acquisition of thermosensitive stochastic sex determination in nematode androdioecious hermaphrodites
Source: Nat Commun. 2024 Nov 7;15:9649. doi: 10.1038/s41467-024-53854-6 (PMC11544036; doi:10.1038/s41467-024-53854-6)
Supplement: Supplementary file 1 — Supplementary Information [file 41467_2024_53854_MOESM1_ESM.pdf]

Rapid chromosome evolution and acquisition of thermosensitive stochastic sex determination in nematode androdioecious hermaphrodites

Kohta Yoshida, Hanh Witte, Ryo Hatashima, Simo Sun, Taisei Kikuchi,
Waltraud Röseler & Ralf J. Sommer

Supplementary Information

Supplementary Tables	2
Supplementary Figures	4

Supplementary Tables

Supplementary Table 1 The number of orthogroup genes previously assigned to Nigon elements in *P. fissidentatus* chromosomes.

	P. fissidentatus chromosomes							Not detected*	% Mode in detected genes
	ChrI	ChrII	ChrIII	ChrIV	ChrV	ChrVI	ChrX		
NigonA	4	5	1	2	423	3	2	89	96%
NigonB	1	264	0	1	1	1	1	57	98%
NigonC	3	3	418	0	0	1	1	91	98%
NigonD	1	1	2	203	1	1	0	63	97%
NigonE	172	0	0	1	0	1	0	66	99%
NigonN	3	0	0	0	0	128	0	41	98%
NigonX	1	0	1	0	0	0	80	37	98%

Orthogroup genes in *C. elegans* genome was used for the analysis. These genes were searched in the list of the one-to-one orthologs between *C. elegans* and *P. fissidentatus* in the present study. *, the number of orthogroup genes not detected in the one-to-one orthologs.

Supplementary Table 2 Sequences of the primers and CRISPR guide RNA

Name of nucleotide sequences	Sequence (5' to 3')
**Primers**	
RH5401	AAAGATTAAGCCATGCATG
RH5403	AGCTGGAATTACCGCGGCTG
Pmay-tra-1.1F	TGCTCGGTCAGTACTAAATCGT
Pmay-tra-1.1R	CCTGTCACTGGCATTGGAGA
**CRISPR guide RNA (site-specific sequence)**	
guide RNA for *Pmay-tra-1.1*	ACGCACATGCGAACCCACAC

Supplementary Figures

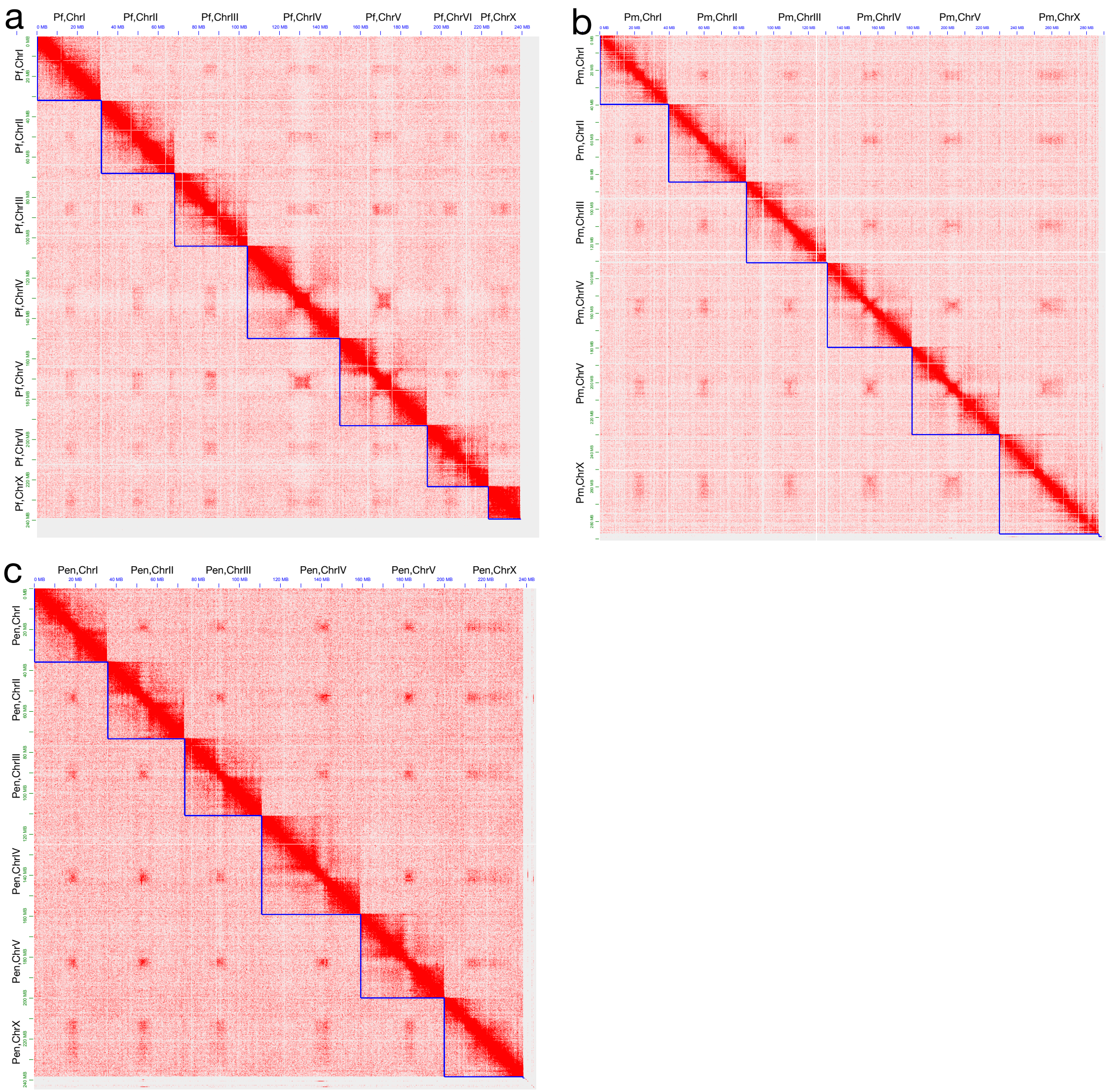

Supplementary Fig. 1 Hi-C contact map of new genome assemblies of (a) *P. fissidentatus*, (b) *P. mayeri* and (c) *P. entomophagus.*
Blue lines indicate resultant scaffolds.

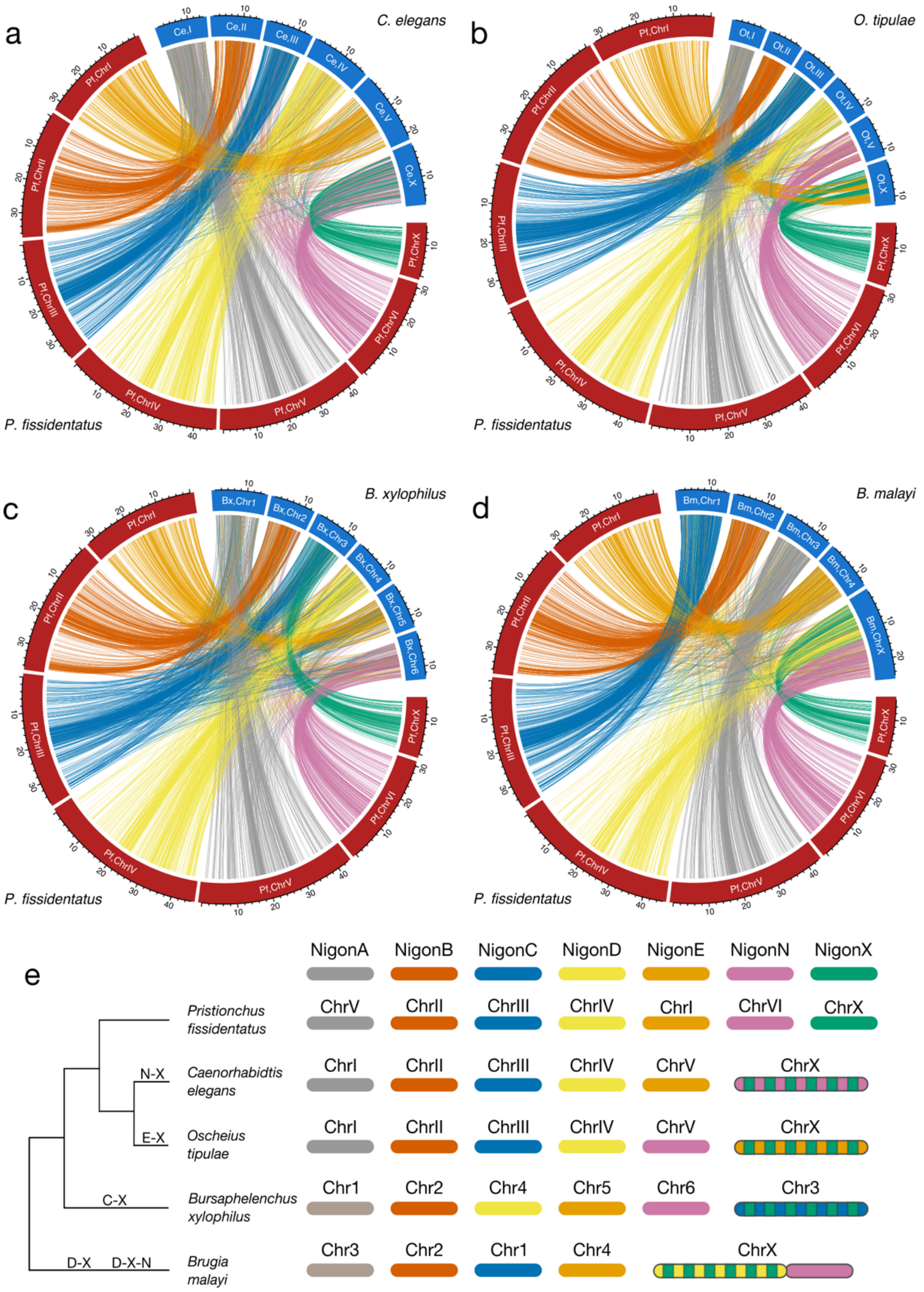

Supplementary Fig. 2 Seven chromosomes of *P. fissidentatus* are conserved chromosome elements across nematodes. a-d. Comparative synteny analysis between *P. fissidentatus* and *Caenorhabditis elegans* (a), *Oscheius tipulae* (b), *Bursaphelenchus xylophilus* (c) *and Brugia malayi* (d), respecitvely. Three thousand pairs of one-to-one ortholog genes were randomly selected for each species pair, and their genomic positions were displayed as links. Colors of links represents *P. fissidentatus* chromosomes. **e.** Conserved Nigon elements and chromosome evolution across nematodes. The previously-described chromosome evolution in the outgroup species was confirmed by the comparative synteny analysis with *P. fissidentatus*, which has conserved ancestral nematode chromosomes.

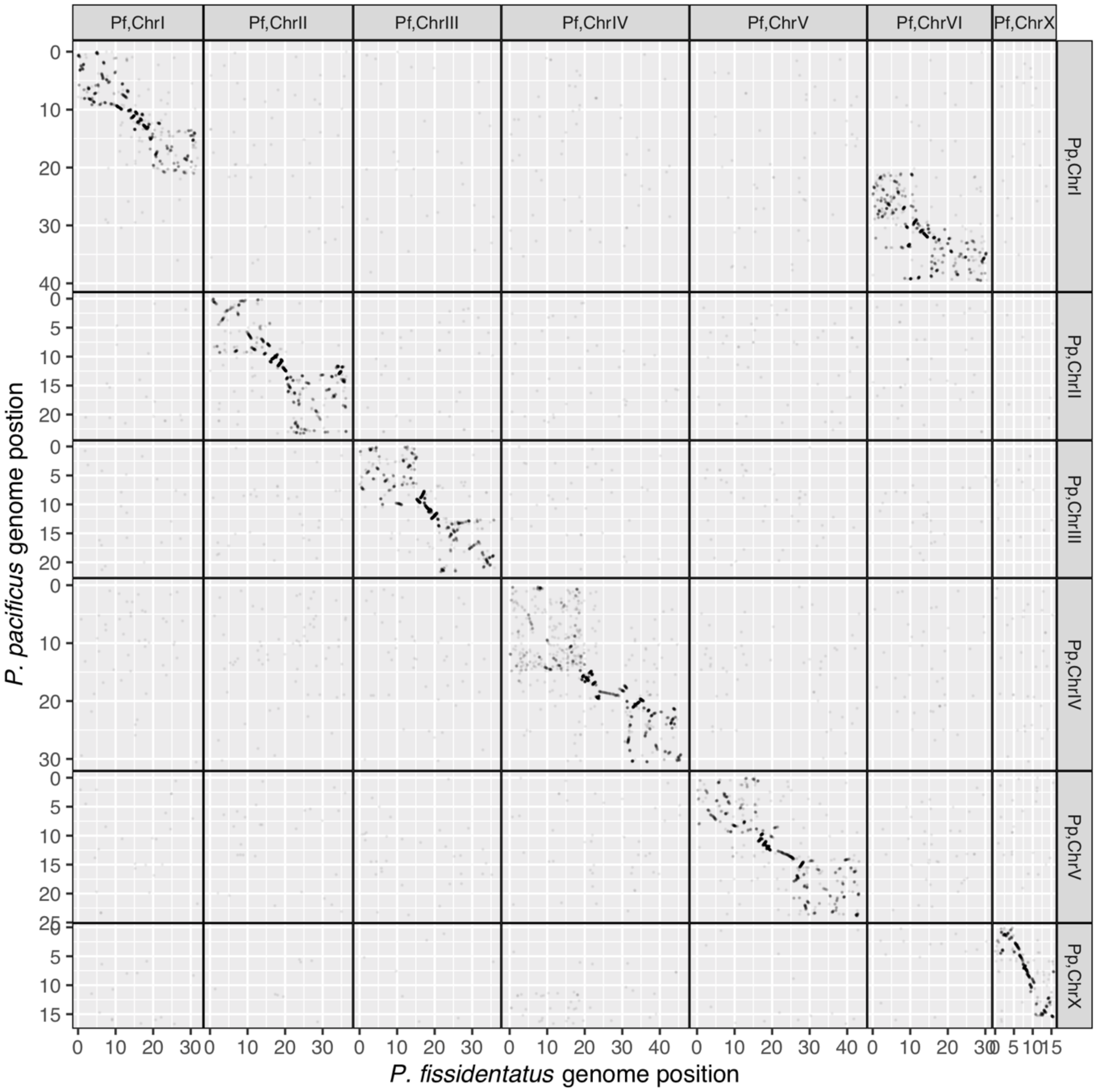

Supplementary Fig. 3 Dot plot analysis of comparative synteny between *P. fissidentatus* and *P. pacificus*.

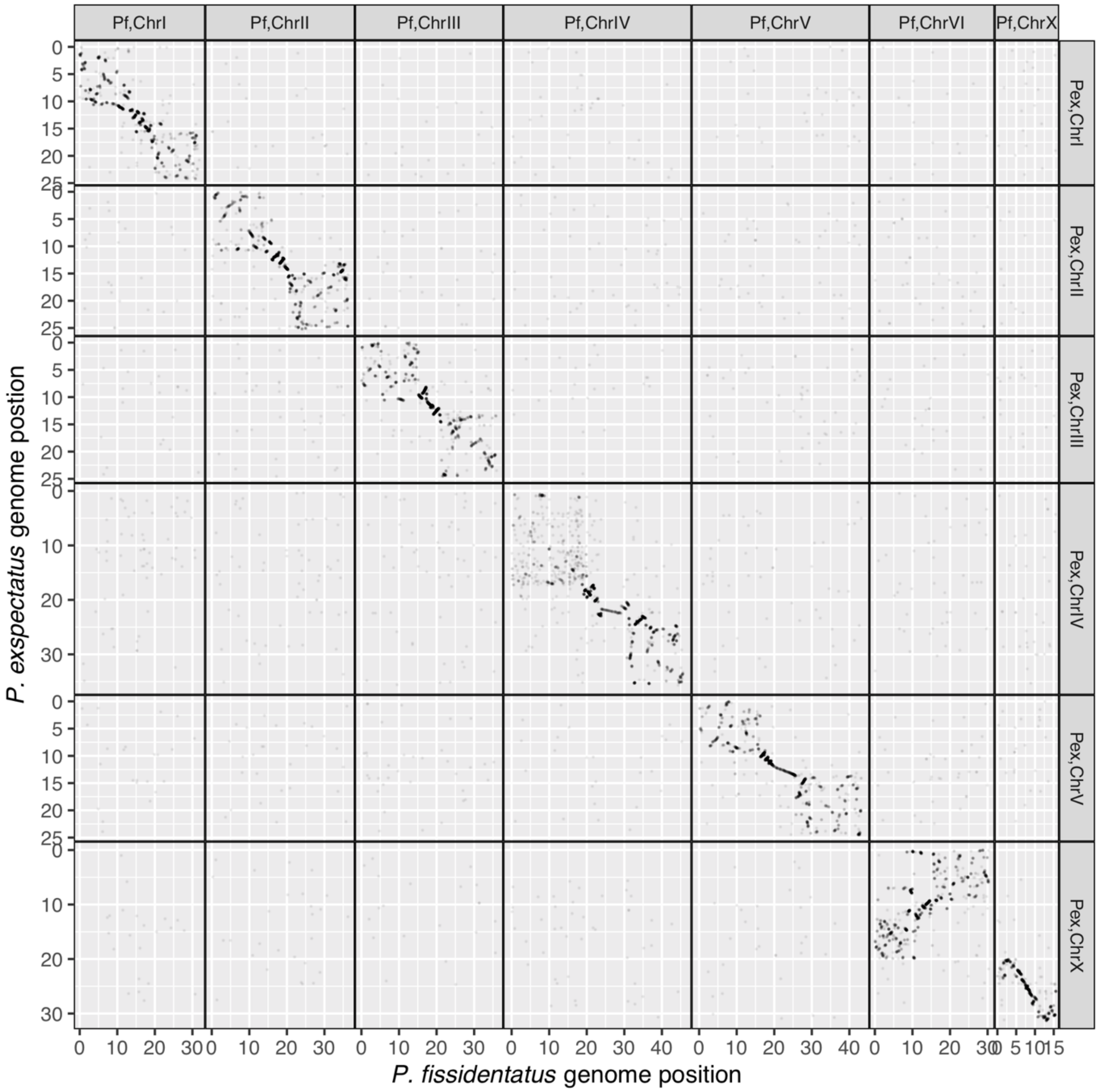

Supplementary Fig. 4 Dot plot analysis of comparative synteny between *P. fissidentatus* and *P. exspectatus*.

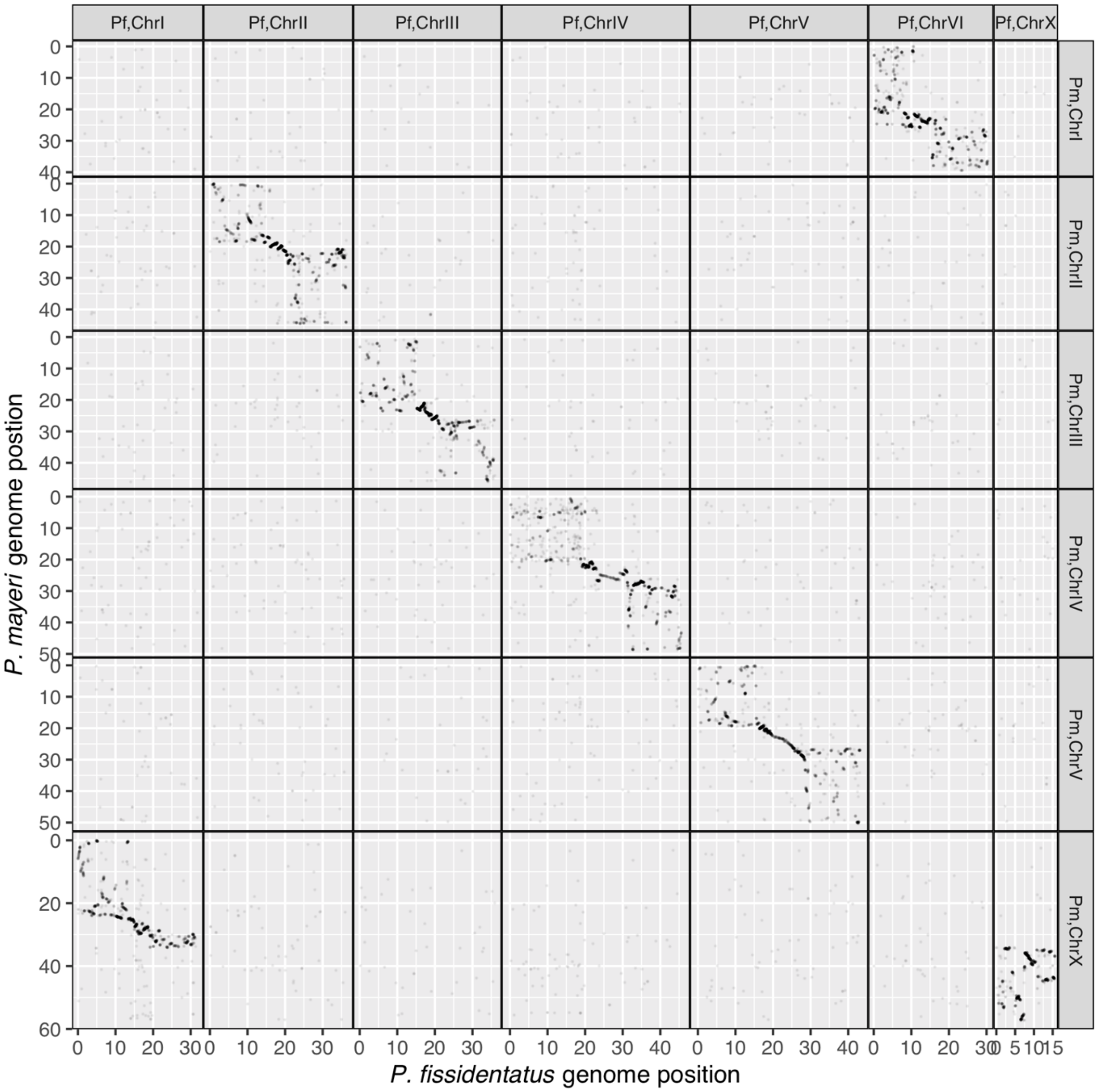

Supplementary Fig. 5 Dot plot analysis of comparative synteny between *P. fissidentatus* and *P. mayeri*.

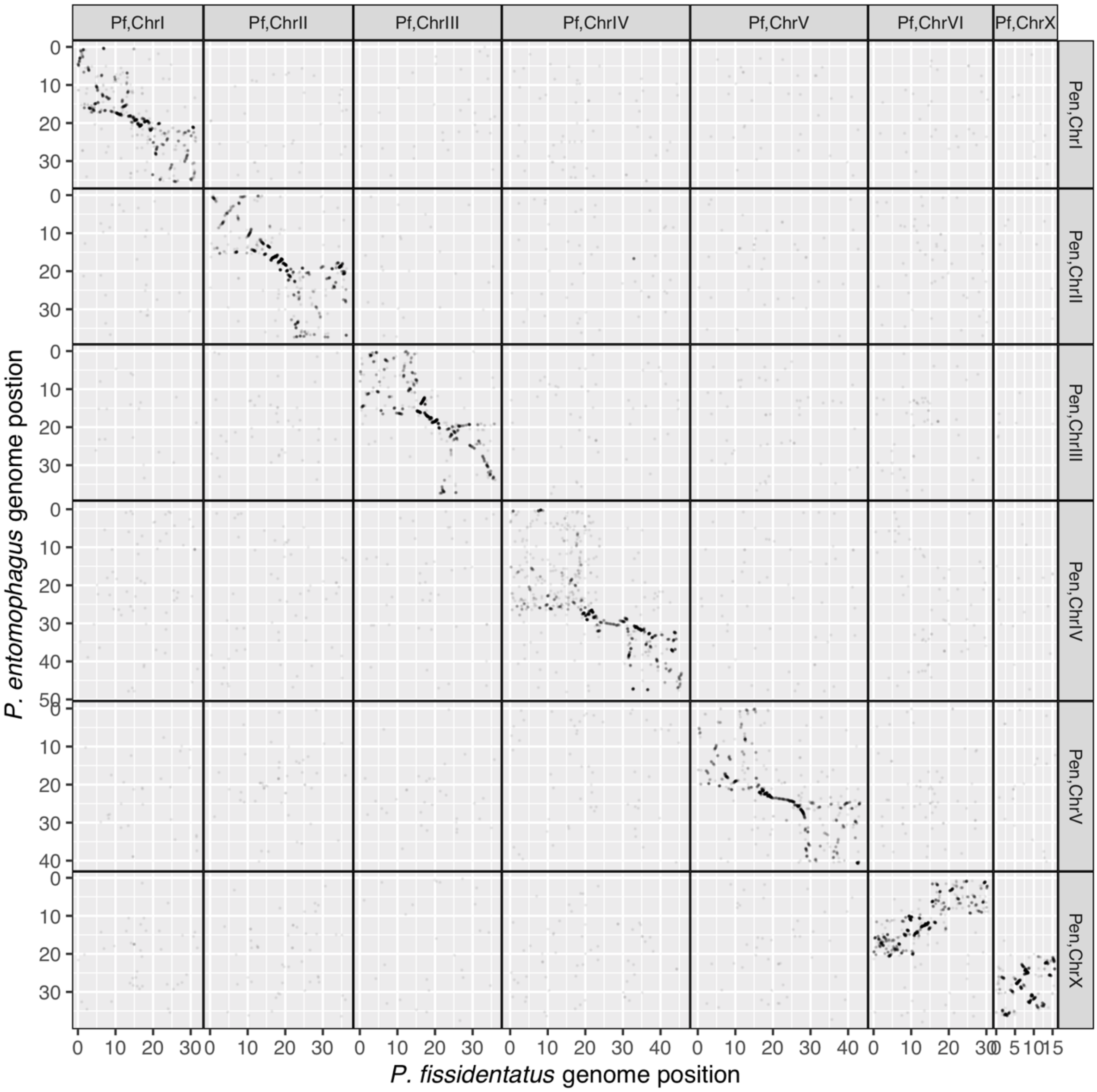

Supplementary Fig. 6 Dot plot analysis of comparative synteny between *P. fissidentatus* and *P. entomophagus.*

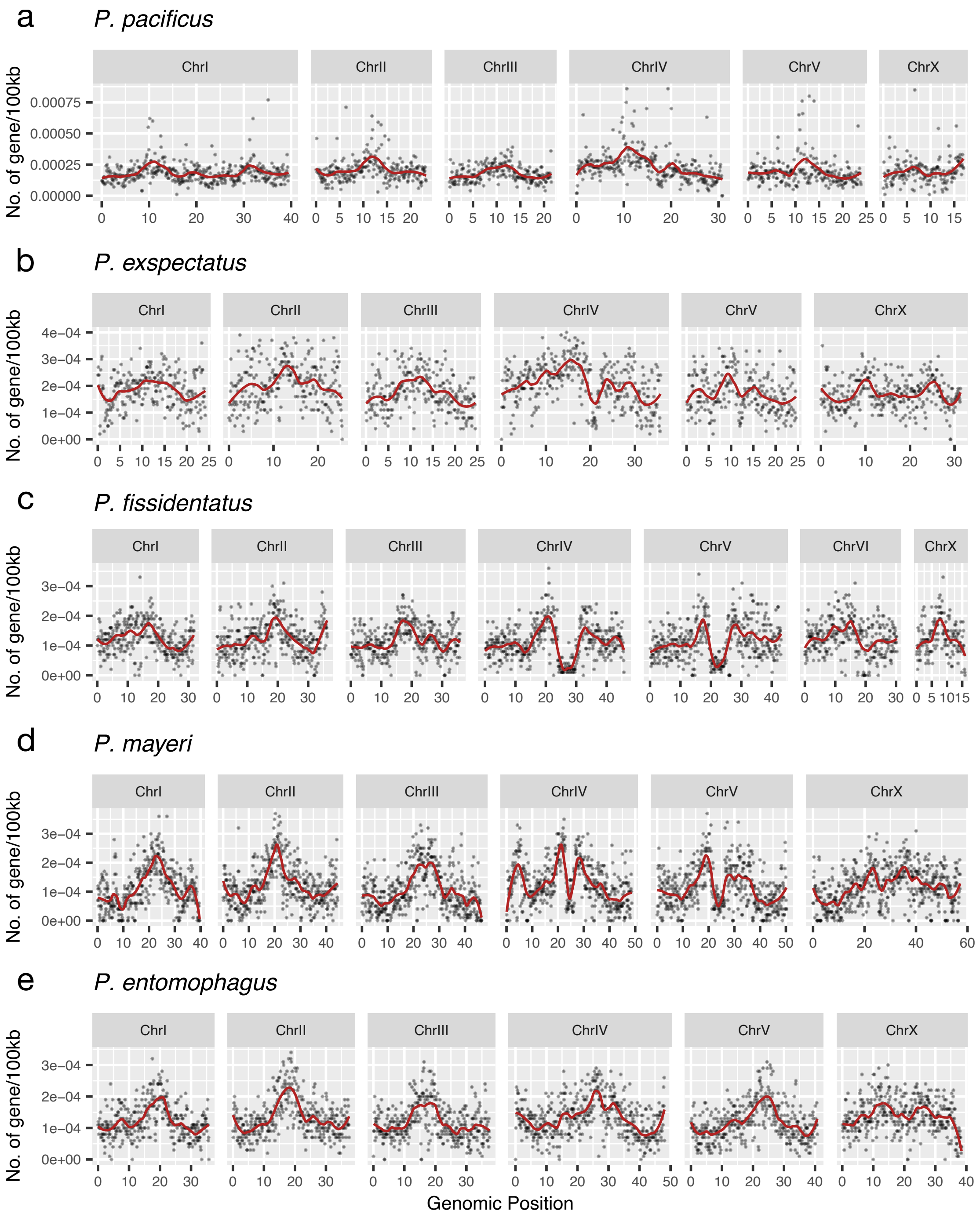

Supplementary Fig. 7 Chromosomal pattern of gene density of five *Pristionchus* species. Gene density of each 100kb sliding window is shown as black points. Red lines indicate LOESS regression lines.
