# Supplementary material for: Rapid chromosome evolution and acquisition of thermosensitive stochastic sex determination in nematode androdioecious hermaphrodites
Source: Nat Commun. 2024 Nov 7;15:9649. doi: 10.1038/s41467-024-53854-6 (PMC11544036; doi:10.1038/s41467-024-53854-6)
Supplement: Supplementary file 2 — Description of Additional Supplementary Files [file 41467_2024_53854_MOESM2_ESM.pdf]

Description of Additional Supplementary Files

File Name: Supplementary Data 1

Description: Karyotyping data of studied strains. The proportions of prophase I or gamete cells that have the chromosome number indicated in the header are shown in the table.

File Name: Supplementary Data 2

Description: Orthofinder results of sex determination genes of *C. elegans*. The number, identifiers and genomic loci of detected ortholog genes are shown. The results for different species are presented on separate sheets.
