# Supplementary material for: Rapid chromosome evolution and acquisition of thermosensitive stochastic sex determination in nematode androdioecious hermaphrodites
Source: Nat Commun. 2024 Nov 7;15:9649. doi: 10.1038/s41467-024-53854-6 (PMC11544036; doi:10.1038/s41467-024-53854-6)
Supplement: Supplementary file 5 — Reporting Summary [file 41467_2024_53854_MOESM5_ESM.pdf]

nature portfolio

Corresponding author(s): Kohta Yoshida
Ralf J. Sommer

Last updated by author(s): Oct 3, 2024

Reporting Summary

Nature Portfolio wishes to improve the reproducibility of the work that we publish. This form provides structure for consistency and transparency in reporting. For further information on Nature Portfolio policies, see our Editorial Policies and the Editorial Policy Checklist.

Statistics

For all statistical analyses, confirm that the following items are present in the figure legend, table legend, main text, or Methods section.

n/a	Confirmed	
☐	☒	The exact sample size (n) for each experimental group/condition, given as a discrete number and unit of measurement
☐	☒	A statement on whether measurements were taken from distinct samples or whether the same sample was measured repeatedly
☐	☒	The statistical test(s) used AND whether they are one- or two-sided<br>*Only common tests should be described solely by name; describe more complex techniques in the Methods section.*
☐	☒	A description of all covariates tested
☐	☒	A description of any assumptions or corrections, such as tests of normality and adjustment for multiple comparisons
☐	☒	A full description of the statistical parameters including central tendency (e.g. means) or other basic estimates (e.g. regression coefficient) AND variation (e.g. standard deviation) or associated estimates of uncertainty (e.g. confidence intervals)
☐	☒	For null hypothesis testing, the test statistic (e.g. F, t, r) with confidence intervals, effect sizes, degrees of freedom and P value noted<br>*Give P values as exact values whenever suitable.*
☒	☐	For Bayesian analysis, information on the choice of priors and Markov chain Monte Carlo settings
☒	☐	For hierarchical and complex designs, identification of the appropriate level for tests and full reporting of outcomes
☐	☒	Estimates of effect sizes (e.g. Cohen's d, Pearson's r), indicating how they were calculated

Our web collection on statistics for biologists contains articles on many of the points above.

Software and code

Policy information about availability of computer code

Data collection	No software or code was used for the data collection in the analysis
Data analysis	Juicer ver. 1.6, 3D-DNA ver. 180114, Juicebox ver. 1.11.08, LASTZ ver. 1.04.00, Perl Packages for Customized Annotation Computing annotation pipe line (PPCAC) ver. 1.0, NCBI BLAST+ ver. 2.14.0, Samtools ver. 1.1.0, jellyfish ver. 2.3.0, Orthofinder ver. 2.5.4. The custom scripts for data analysis are available in https://figshare.com/projects/Datasets_and_scripts_for_P_mayeri_sex_determination_project/178452.

For manuscripts utilizing custom algorithms or software that are central to the research but not yet described in published literature, software must be made available to editors and reviewers. We strongly encourage code deposition in a community repository (e.g. GitHub). See the Nature Portfolio guidelines for submitting code & software for further information.

Data

Policy information about availability of data

All manuscripts must include a data availability statement. This statement should provide the following information, where applicable:

- Accession codes, unique identifiers, or web links for publicly available datasets
- A description of any restrictions on data availability
- For clinical datasets or third party data, please ensure that the statement adheres to our policy

Genome assembly generated in this study and raw sequence reads are available in NCBI or DDBJ (Project accessions: PRJDB16629- PRJDB16631, PRJDB16724-PRJDB16728; Assembled Data Accessions: BTSK01000001-BTSK01000073, BTSX01000001-BTSX01000172 and BTSY01000001-BTSY01000372) or https://

pristionchus.org/download (only assembly and annotation). Microscopy data reported in this paper will be shared by the lead contact upon request. The other raw data are available in https://figshare.com/projects/Datasets_and_scripts_for_P_mayeri_sex_determination_project/178452.

Research involving human participants, their data, or biological material

Policy information about studies with human participants or human data. See also policy information about sex, gender (identity/presentation), and sexual orientation and race, ethnicity and racism.

Reporting on sex and gender	n/a
Reporting on race, ethnicity, or other socially relevant groupings	n/a
Population characteristics	n/a
Recruitment	n/a
Ethics oversight	n/a

Note that full information on the approval of the study protocol must also be provided in the manuscript.

Field-specific reporting

Please select the one below that is the best fit for your research. If you are not sure, read the appropriate sections before making your selection.

[x] Life sciences   [ ] Behavioural & social sciences   [ ] Ecological, evolutionary & environmental sciences

For a reference copy of the document with all sections, see nature.com/documents/nr-reporting-summary-flat.pdf

Life sciences study design

All studies must disclose on these points even when the disclosure is negative.

Sample size	The near-largest number of the samples that can be handled at the same time is used.
Data exclusions	No data was excluded.
Replication	In the experimental result of sex ratio, different strains or different mutants indicated replicable results.
Randomization	The comparative test was done with the same clonal population removing genetic effects.
Blinding	The blinding was not capable due to the experimental setup.

Reporting for specific materials, systems and methods

We require information from authors about some types of materials, experimental systems and methods used in many studies. Here, indicate whether each material, system or method listed is relevant to your study. If you are not sure if a list item applies to your research, read the appropriate section before selecting a response.

Materials & experimental systems

n/a	Involved in the study	
[x]	[ ]	Antibodies
[x]	[ ]	Eukaryotic cell lines
[x]	[ ]	Palaeontology and archaeology
[ ]	[x]	Animals and other organisms
[x]	[ ]	Clinical data
[x]	[ ]	Dual use research of concern
[x]	[ ]	Plants

Methods

n/a	Involved in the study	
[x]	[ ]	ChIP-seq
[x]	[ ]	Flow cytometry
[x]	[ ]	MRI-based neuroimaging

Animals and other research organisms

Policy information about studies involving animals; ARRIVE guidelines recommended for reporting animal research, and Sex and Gender in Research

Laboratory animals	Pristionchus pacificus, PS312; Pristionchus exspectatus, RS5522B; Pristionchus occultus, RS5811B1; Pristionchus sikae, RS5901B2;

	Pristionchus arcanus, RS5527; Pristionchus kurosawai, RS5914; Pristionchus taiwanensis, RS5797; Pristionchus maxplancki, RS5594; Pristionchus laevicolis, RS5939; Pristionchus japonicus, RS5238; Pristionchus neolucani, RS5949; Pristionchus riukiariae, RS5937; Pristionchus degawai, RS5938; Pristionchus dorci, RS6134; Pristionchus hongkongensis, RS5957; Pristionchus quartusdecimus, RS5230; Pristionchus purgamentorium, RS6138; Pristionchus marianneae, RS5108; Pristionchus aerivorus, RS5106; Pristionchus maupasi, RS0143; Pristionchus americanus, RS5140; Pristionchus boliviae, RS5262; Pristionchus mayeri, RS5460, RSA035, RS4203 (tu1863), RS4204 (tu1864), RS4205 (tu1865); Pristionchus atlanticus, CZ3975; Pristionchus pauli, RS5151; Pristionchus brevicauda, RS5231; Pristionchus clavus, RS5284; Pristionchus uniformis, RS0141; Pristionchus bulgaricus, RS5283; Pristionchus entomophagus, RS0144; Pristionchus lheritieri, SB245; Pristionchus lucani, RS5050; Pristionchus hoplostomus, JU1090; Pristionchus musae, RS5987; Pristionchus auriculiariae, RS5989; Pristionchus fukushimae, RS5595; Pristionchus triformis, RS5233; Pristionchus magnoliae, RS5999; Different sexes and ages are used depending on the experiments.
Wild animals	No wild animals
Reporting on sex	Sex of the samples in each experiment are described in the manuscript.
Field-collected samples	No field-collected samples
Ethics oversight	Nematodes are not included in the animal welfare act of Germany or Japan. No ethical approval or guidance was required.

Note that full information on the approval of the study protocol must also be provided in the manuscript.

Plants

Seed stocks	n/a
Novel plant genotypes	n/a
Authentication	n/a
